# Rorschach test with exner cs in assessing damage and trauma in suspected cases of abuse. Traumatic intrusions in thinking: Ptsd and adaptation disorder

**DOI:** 10.1192/j.eurpsy.2021.1210

**Published:** 2021-08-13

**Authors:** D. Galletta, V. Suarato, M. Confuorto, F. Subosco, A.M. Mastrola

**Affiliations:** Department Of Head-neck Care Unit Of Psychiatry And Psychology “federico Ii” University Hospital Naples, “Federico II” University Hospital Naples, Italy, Naples, Italy

**Keywords:** abuse, damage assessment, Rorschach, adaptation disorder

## Abstract

**Introduction:**

This study wants to identify elements that could be informative in diagnosis and prognosis process of all those subjects who, following traumatic experiences, may develop PTSD, or even show signs of a more general and pervasive adaptation disorder, allowing a more precise damage assessment.

**Objectives:**

In this perspective, the analysis of the Rorschach test according to the comprehensive system of Exner, reading Structural Summary and the analysis of the constellations, allows to make interesting inferences, in all the descriptive areas associated with the key variables as regards not only the cognitive area (Processing >> Mediation >> Ideation) but also the affective and relational area (Interpersonal Perception >> Self-Perception >> Controll >> Affect), so as to have a picture of the functioning of these subjects and, to be able to plan a more functional therapeutic plan.

**Methods:**

It is based on a sample of 29 subjects, 20 women and 9 men with an average age of about 35 years (54-14 years), who came to the attention of the clinic, at the request of the reference psychiatrist for diagnostic personality assessment. All subjects complained of various kinds of discomfort, affective-relational difficulties and anxious-depressive symptoms.

**Results:**

The results that emerged, in line with the initial hypotheses, converge in describing a personality style, not very resilient that could suffer in overcoming difficulties and in the search for new equilibrium.

**Conclusions:**

It’s emphasized how the weight of a traumatic event like abuse can evolve into an adaptation disorder,strongly affecting the functionality of the subjects and their social integration.

